# Dopamine and Pain Sensitivity: Neither Sulpiride nor Acute Phenylalanine and Tyrosine Depletion Have Effects on Thermal Pain Sensations in Healthy Volunteers

**DOI:** 10.1371/journal.pone.0080766

**Published:** 2013-11-13

**Authors:** Susanne Becker, Marta Ceko, Mytsumi Louis-Foster, Nathaniel M. Elfassy, Marco Leyton, Yoram Shir, Petra Schweinhardt

**Affiliations:** 1 Alan Edwards Centre for Research on Pain and Faculty of Dentistry, McGill University, Montreal, Quebec, Canada; 2 Department of Cognitive and Clinical Neuroscience, Central Institute of Mental Health, Medical Faculty Mannheim, Heidelberg University, Mannheim, Germany; 3 National Center for Complementary and Alternative Medicine, National Institutes of Health, Bethesda, Maryland, United States of America; 4 Department of Neurology and Neurosurgery, Faculty of Medicine, McGill University, Montreal, Quebec, Canada; 5 Department of Psychiatry, McGill University, Montreal, Quebec, Canada; 6 Center for Studies in Behavioral Neurobiology, Concordia University, Montreal, Quebec, Canada; 7 Alan Edwards Pain Management Unit, Montreal General Hospital, McGill University Health Centre, Montreal, Quebec, Canada; University of Arizona, United States of America

## Abstract

Based on animal studies and some indirect clinical evidence, dopamine has been suggested to have anti-nociceptive effects. Here, we investigated directly the effects of increased and decreased availability of extracellular dopamine on pain perception in healthy volunteers. In Study 1, participants ingested, in separate sessions, a placebo and a low dose of the centrally acting D2-receptor antagonist sulpiride, intended to increase synaptic dopamine via predominant pre-synaptic blockade. No effects were seen on thermal pain thresholds, tolerance, or temporal summation. Study 2 used the acute phenylalanine and tyrosine depletion (APTD) method to transiently decrease dopamine availability. In one session participants ingested a mixture that depletes the dopamine amino acid precursors, phenylalanine and tyrosine. In the other session they ingested a nutritionally balanced control mixture. APTD led to a small mood-lowering response following aversive thermal stimulation, but had no effects on the perception of cold, warm, or pain stimuli. In both studies the experimental manipulation of dopaminergic neurotransmission was successful as indicated by manipulation checks. The results contradict proposals that dopamine has direct anti-nociceptive effects in acute experimental pain. Based on dopamine’s well-known role in reward processing, we hypothesize that also in the context of pain, dopamine acts on stimulus salience and might play a role in the initiation of avoidance behavior rather than having direct antinociceptive effects in acute experimental pain.

## Introduction

Dopamine has well described and oft-cited roles in motivational states, reward processing, and motor functions (see 1,2 for review). But dopamine also plays a role in the processing of nociceptive stimuli. Specifically, it has been hypothesized that dopamine has direct anti-nociceptive effects [3-7]. This hypothesis is based on three main lines of evidence: rodent studies, clinical data, and genetic associations. Rodent studies, typically using interventions such as intrathecal or intracerebral microinjections of receptor a- and antagonists, indicate that effects of dopamine on nociceptive processing are mainly mediated by striatal dopaminergic D2-receptors (e.g. [8-10]). In contrast, activation or inhibition of striatal D1-receptors by microinjections of receptor agonists or antagonists has no effects on nociceptive processing [8-10]. Anti-nociceptive effects of D2-receptor activation and pro-nociceptive effects of D2-receptor inhibition have been shown in tonic pain models such as the writhing and formalin tests, in deafferentation and neuropathic pain models (e.g. [11-14]), and in phasic pain models using thermal and mechanical stimuli, including the tail flick, hot plate or paw pressure test (e.g. [9,14,15]). Further, it has been reported that D2-receptor activation is important for endogenous pain inhibition through descending pathways [16,17]. In line with these results, D2-receptor activation has been shown to inhibit spinal wind-up induced by electrical stimulation [17] and anti-nociceptive actions on spinal cord neurons in superficial laminae through descending dopaminergic pathways have been shown in *in vivo* patch-clamp studies [18].

Clinically, it has been observed that dopamine agonists can alleviate pain [19,20] and striatal dopaminergic neurotransmission has been found to be altered in chronic pain syndromes such as fibromyalgia, burning mouth, and atypical facial pain [21-23]. In addition, patients with Parkinson’s disease, which involves massive loss of dopaminergic neurons in the substantia nigra, can present with augmented pain perception (e.g. [24,25]). Further, genetic associations have been reported between experimental and clinical pain and dopamine-related genes [26,27], for example relationships between cold pain tolerance and dopamine transporter (DAT-1) as well as monoamine oxidase-A (MAO-A) polymorphisms [26].

Despite the wealth of data, in particular from animal studies, suggesting an effect of dopamine on pain processing, this has only rarely been directly tested in humans in either experimental or clinical pain. In the available studies [28,29], the dopamine receptor agonist apomorphine has been reported to affect cold pain tolerance and conditioned pain modulation but not cold and thermal pain thresholds or the perception of suprathreshold heat pain stimuli in healthy volunteers [28,29]. The latter results are in contrast to the animal literature, which suggests antinociceptive effects of dopamine also on phasic stimuli and thresholds. We submit therefore that more studies are needed to clarify dopamine’s role in the processing of nociceptive stimuli. 

The aim of our two studies was to investigate whether dopamine directly affects pain perception in healthy volunteers. In the first study we used a low dose of the D2-receptor antagonist sulpiride because low doses of sulpiride lead to increased dopamine release through predominant pre-synaptic effects (e.g. [30-33]; see 34 for review). We tested whether increased availability of dopamine has hypoalgesic effects, assessed with thermal pain thresholds, pain tolerance, temporal summation, and perceived unpleasantness of thermal stimuli close the pain threshold. Because ceiling effects could possibly mask effects of increased dopamine on pain perception, we tested in a second study using the acute phenylalanine and tyrosine depletion method whether decreased cerebral availability of dopamine increases the perceived intensity and unpleasantness of heat pain stimuli.

## Methods: General

The studies were approved by the McGill University Institutional Review Board and written informed consent was obtained from all participants according to the revised Declaration of Helsinki. Exclusion criteria were any reported present or past pain conditions, psychiatric disorders, excessive gambling, substance abuse behaviors, alcohol consumption of more than 100 ml alcohol per week, habitual consumption of recreational drugs, tobacco use, regular or frequent night shifts or sleep disorders.

## Methods Study 1: Central Pre-Synaptic D2-Receptor Blockade

### Participants

Twenty-four healthy volunteers (11 female, 13 male; age *M*=23.1 yrs, *SD*=5.6 yrs) participated in Study 1. A priori sample size calculation revealed a necessary sample size of 24 usable datasets for detecting a medium effect for the pre-synaptic D2-receptor blockade with a 5% probability of committing a Type 1 error (alpha=0.95), and a 20% probability of committing a Type 2 error (β=0.80).

### General design

**Figure 1:**
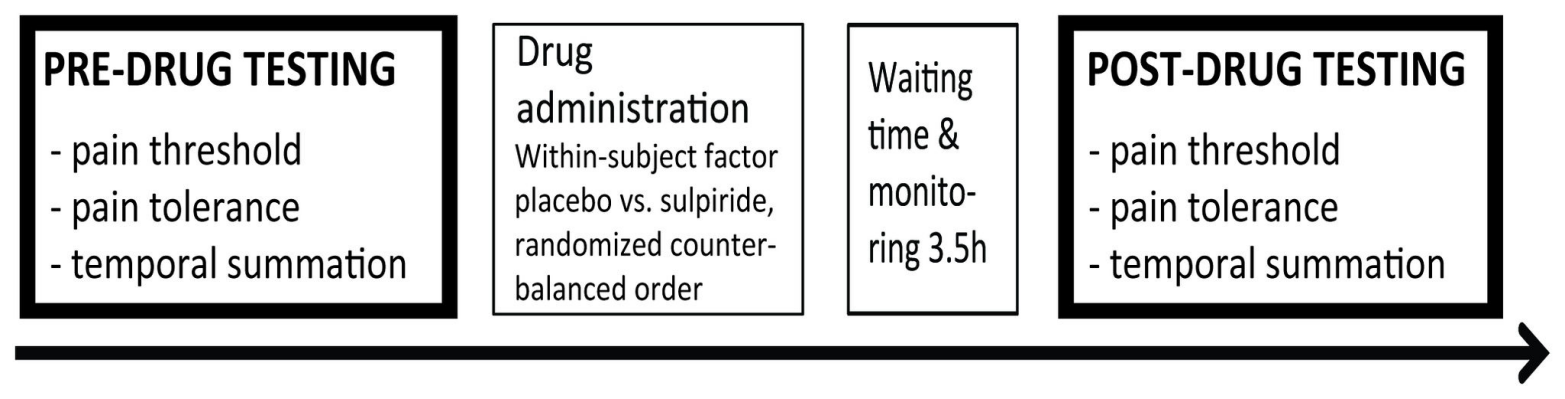
Overview of the time course of an experimental session of Study 1. All participants performed two sessions: in one session they ingested 600 mg sulpiride, in the other one a placebo. In each session, participants performed pre- and post-drug testing, separated by a 3.5h waiting period.

Study 1 consisted of two sessions per participant, at least three days apart (*M* = 7.9 days, *SD* = 4.64 days), given in a double-blind, counter-balanced design. In both sessions, pre-drug testing was performed, then participants ingested the drug (sulpiride or placebo) and 3.5 hours after drug intake, post-drug testing was performed to investigate the effect of sulpiride on pain sensitivity. See Figure 1 for an overview of the experimental design of Study 1.

Before starting the experiments, participants were familiarized with the thermal stimuli and the testing procedures, and were trained to use the rating scales appropriately.

### Thermal stimulation

Heat stimuli were applied using a 27 mm diameter contact thermode (Contact Heat Evoked Potentials, CHEPS; PATHWAY Pain & Sensory Evaluation System, Medoc Ltd. Advanced Medical System, Israel). Baseline temperature was 32°C. For safety reasons, temperatures above 50°C were not allowed. Thermal stimuli were applied to the thenar eminence of participants’ non-dominant hand. Thenar stimulation was achieved by participants placing their palm on the thermode that was embedded in a hemisphere made of Styrofoam®.

### Rating scales

To assess temporal summation, participants rated perceived intensity of the thermal stimuli on a horizontally orientated Visual Analogue Scale (VAS). The intensity VAS ranged from 0 “no sensation” to 200 “most intense pain tolerable” with 100 being the pain threshold. The pleasantness/unpleasantness VAS ranged from -100 “extremely unpleasant” to +100 “extremely pleasant” with the midpoint “neutral” [35,36]. 

### Assessment of pain sensitivity

Pain thresholds and tolerance were assessed with the methods of limits, with three stimuli increasing in temperature at a rate of 1.5°C/s until the participant felt the slightest pain sensation (pain threshold) or could not tolerate the stimulus anymore (pain tolerance). The participant indicated that the threshold/tolerance level was reached by pressing a mouse button after which the temperature of the thermode returned immediately to the baseline temperature. Temporal summation was assessed by the application of ten one-second long stimuli of 49°C at a frequency of 0.3 Hz [37] after a single presentation of an identical stimulus. Temporal summation was measured as the difference between the maximum intensity rating for the ten stimuli and the single stimulus [38].

For the assessment of perceived unpleasantness of thermal stimuli close to the pain threshold, participants first received an ascending series of thermal stimuli (starting at 41°C, increments of 1°C), which they rated on the intensity and unpleasantness VASs. This series was stopped when participants rated the stimulus as painful (100 or above on the intensity rating scale). Implementing a staircase method, participants received thermal stimuli until their intensity ratings were stable, i.e. when the same applied temperature was at least twice rated as the pain threshold (±5 points on the intensity scale).

### D2-receptor blockade

Participants ingested a low dose of the D2-receptor antagonist sulpiride (600mg, p.o., [39]) in one testing session and a placebo (microcrystalline cellulose) in the other. Low doses of sulpiride act predominately at pre-synaptic sites, blocking D2-autoreceptors, suppressing negative feedback, and as a result, potentiating release of phasic dopamine in response to environmental stimuli [40]. The predominate pre-synaptic action of sulpiride (see 34 for review) has been demonstrated by animal studies showing that sulpiride antagonizes pre-synaptic effects of low dose apomorphine such as inhibition of amphetamine-hyperactivity as well as decrease of dopamine turnover [30,31,41]. Further, *in vivo* microdialysis studies demonstrate increased dopamine release induced by sulpiride in the striatum and medial prefrontal cortex [32,42]. In humans, the notion of predominant pre-synaptic effects of sulpiride is supported by antidepressive effects at low doses of sulpiride [33,43]. 

The effectiveness of a single dose of 600 mg sulpiride was investigated in a pilot study with ten healthy volunteers (5 female, 5 male; age *M* = 24.70 yrs, *SD* = 3.40 yrs; testing at four time points: before ingestion, 2h, 4h, and 5h after ingestion), testing for deteriorating effects on spatial working memory that have been reported before with single doses of 400 mg sulpiride [44,45].

After drug intake, participants waited for 3.5 hours during which they studied, read, or watched movies. We ensured that the emotional content of the media participants were exposed to during the waiting period was low to avoid effects on pain ratings in the post-drug testing. During the waiting period participants were asked every 30 minutes to rate potential side effects (from 0 ‘not at all’ to 5 ‘severe’) from a provided list that included possible side effects of sulpiride as well as changes in subjective well-being. Blood pressure was measured at the same time points. The 3.5 hours waiting time was chosen based on peak plasma concentrations of sulpiride reported to be 3.5 to 4.5 hours after ingestion [46] and the occurrence of central effects [47]. At the end of each testing session, participants as well as the experimenter indicated whether they thought the participant received the placebo or the drug (exit interview, response alternatives: ‘placebo’, ‘drug’, or ‘don’t know’) to test for potential unblinding.

### Statistical analysis

To test the effects of the D2-receptor blockade, pain thresholds, pain tolerance, temporal summation, and unpleasantness ratings of stimuli close to the pain threshold implemented in the staircase method were analyzed, after confirming normality (Shapiro-Wilk test), with a repeated measurement ANOVA design using mixed model procedures with the factors ‘drug’ (with the levels sulpiride and placebo) and ‘testing’ (with the levels pre- and post-drug). 

The effects of sulpiride on spatial working memory (pilot study), blood pressure, side effects, and answers in the exit interview were analyzed. Spatial errors in the spatial working memory were analyzed with an ANOVA design by mixed model procedures, with the two within-subjects factors ‘drug’ (with the levels sulpiride and placebo) and ‘time’ (with the measurement times points before ingestion, 2h, 4h, and 5h after ingestion). Blood pressure data were analyzed with an ANOVA design by mixed model procedures, with the two within-subjects factors ‘drug’ (with the levels sulpiride and placebo) and ‘time’ (with the measurement times points 30, 60, 90, 120, 150, 180, 210 min after drug intake and at the end of testing). Side effects ratings between placebo and sulpiride sessions were compared using Wilcoxon Sign Rank tests. Answers in the exit interview were compared to the actual order of sulpiride and placebo intake with χ^2^ tests. 

ANOVA analyses were followed by post-hoc pairwise comparisons, calculation of generalized omega squared (ω^2^) statistics as an unbiased measure of effect sizes in ANOVA designs [48], and statistical power (1-β) [49], when appropriate. Negative values for ω^2^ squared are treated as 0 because negative variance estimates have no meaning [50]; a value of 0.04 is the recommended minimum effect size for ω^2^, 0.25 represents moderate effects and 0.64 strong effects 

[51]. The significance level was set to 5%. All statistical analyses were performed using PASW Statistics 17 (SPSS Inc. Chicago, USA).

## Results Study 1: Central Pre-Synaptic D2-Receptor Blockade

### Manipulation check and side effects of sulpiride

A single dose of 600 mg p.o. sulpiride increased spatial errors in a spatial working memory task compared to placebo (main effect ‘drug’ F_9_=48.67, *p*<0.01, ω^2^=0.64, 1-β=0.99) four hours after drug intake (*p*=0.04; before, 2h and 5h after ingestion: all *p*’s>0.10) in the pilot study, similar to previous publications [44,45].

In line with earlier findings [52-54], sulpiride lowered blood pressure compared to placebo (diastolic blood pressure, main effect ‘drug’ F_344_=57.73, *p*=0.01, ω^2^=0.16, 1-β=0.78) 60 min after drug intake with effects persisting until the end of the post-drug testing (post-hoc comparisons, 60 min: *p*=0.04; 120 min: *p*=0.05; 90, 150, 180, 210 min, after post-drug testing: *p*<0.01).

Participants reported only few mild side effects that did not differ under sulpiride compared to placebo (χ^2^ tests for all side effects: p’s>0.10). Participants and experimenter remained blinded as indicated by the results of the exit interview (χ^2^ tests: participants and experimenter, Day 1 & 2 all *p*’*s*>0.10).

### Sulpiride had no effect on pain sensitivity

For thermal pain threshold, tolerance, and temporal summation the interaction between ‘drug’ and ‘testing’ was not significant (pain threshold F_69_=0.41, *p*=0.84, ω^2^=0, 1-β=0.06; pain tolerance F_69_=0.10, *p*=0.75, ω^2^=0, 1-β=0.07; temporal summation F_68_=1.61, *p*=0.21, ω^2^=0, 1-β=0.40), indicating that sulpiride had no effect on pain sensitivity. Similarly, neither unpleasantness ratings of stimuli near the pain threshold nor the corresponding temperatures were different between sulpiride and placebo (interaction ‘drug’ x ‘testing’ unpleasantness: F_23_=0.02, *p*=0.89, ω^2^=0, 1-β=0.05; temperatures: F_*23*_=0.16, *p*=0.69, ω^2^=0, 1-β=0.07).

## Methods Study 2: Acute Phenylalanine and Tyrosine Depletion

### Participants

Twenty-eight healthy male volunteers (age *M*=21.8 yrs, *SD*=2.0 yrs) participated in Study 2, following the same sample size calculation as in Study 1.

### General design

**Figure 2:**
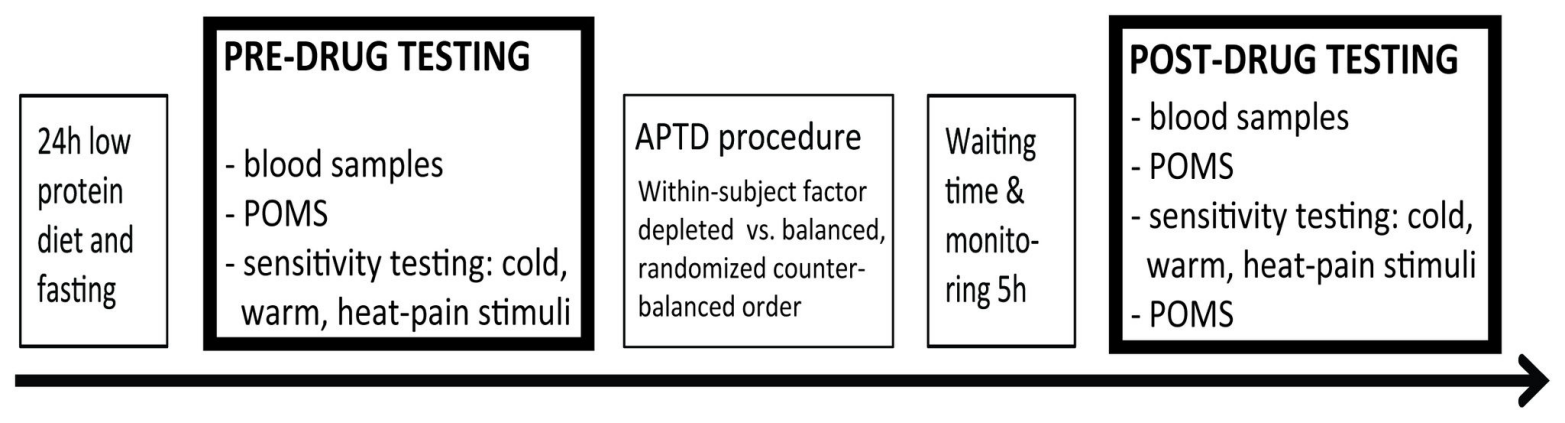
Overview of the time course of an experimental session of Study 2. All participants performed two sessions. In both sessions, participants ingested encapsulated amino acids and an amino acid mixture; in one session this mixture did not contain phenylalanine and tyrosine (depleted) while in the other session it contained these amino acids (balanced) as a control condition. In each session, participants performed pre- and post-ingestion testing, separated by a 5h waiting period.

Participants underwent three sessions. The first session was a familiarization session, which served to explain the experiment and the APTD procedure, familiarize participants with the thermal stimuli and testing procedures, and to train them to use the ratings scales appropriately. The subsequent two experimental sessions, performed one week apart, double-blind and counterbalanced, were identical except for the amino acid mixture given (see below). On the day before each experimental session, participants followed a low-protein diet and fasted from midnight onwards. Participants’ pain and thermal sensitivity was assessed before and five hours after ingestion of the amino acid mixtures (depleted vs. balanced). See Figure 2 for an overview of the experimental design of Study 2.

### Thermal stimuli

Participants’ sensitivity to non-painful cool and warm as well as painful heat stimuli was assessed before and 5 hours after the APTD procedure. The cool and warm stimuli were included as control stimuli to test for unspecific effects of dopamine depletion. Thermal stimuli were applied using a 9 cm^2^ contact thermode (TSA II Neuroanalyzer, Medoc Ltd. Advanced Medical System, Israel). Baseline temperature was 32°C and stimuli either increased or decreased from this baseline at a rate of 10 °C/s. Stimuli were applied to three different sites on participants’ volar forearm. The experimenter applied the stimuli manually, moving from proximal to distal to avoid repeating stimulation of the same site in consecutive trials. For safety reasons, temperatures above 50°C were not allowed. At each testing time point (before and after the APTD procedure), participants received three cool, three warm, and three pain stimuli in pseudo-random order that was the same for every testing time point and every participant. Each stimulus had a plateau of 5 seconds. The temperatures for cool stimuli were 26 °C and 30 °C, 34°C and 36 °C for warm stimuli and either 46 °C or 47.5 °C for pain stimuli. Participants who rated 46 °C between 150 and 170 on the intensity rating scale (see below) during the familiarization session were tested with this temperature, those rating it below 150 but above 100 were tested with 47.5 °C. Participants gave ratings on all four rating scales (see below) immediately after each thermal stimulus.

### Rating scales

Following each thermal stimulus, participants rated perceived intensity and unpleasantness/pleasantness of the thermal stimulus as well as their mood and anxiety/calmness on horizontally orientated VASs. The intensity VAS ranged from 0 “no sensation” to 200 “most intense pain tolerable” with 100 being the pain threshold. The pleasantness/unpleasantness, mood, and anxiety/calmness VASs ranged from -100 “extremely unpleasant/bad/anxious” to +100 “extremely pleasant/good/clam” with the midpoint “neutral” [35,36]. 

### Acute phenylalanine and tyrosine depletion

An established dietary acute phenylalanine and tyrosine depletion (APTD) procedure was used to decrease brain dopamine levels [35]. The synthesis of dopamine depends upon the availability of the amino acid precursors phenylalanine and tyrosine. By administering an amino acid mixture deficient in the precursors, brain dopamine levels can be lowered. The identical APTD procedure has been successfully used before in e.g. studies of emotion-based decision-making and perceptual timing [55,56]. 

At the two testing days, participants ingested encapsulated amino acids and one of two amino acid mixtures dissolved in chocolate milk or orange juice. One of the mixtures did not contain phenylalanine and tyrosine (depleted) while the other contained these amino acids (balanced) as a control condition. Composition, preparation and administration of the mixtures was based on established procedures [57,58]. Microdialysis [59], neuroendocrine [60], and PET [^11^C]raclopride [61,62] studies all suggest that APTD affects dopamine release, with clear effects in humans 5 hours after ingestion [61,62]. Therefore, a waiting time of 5 hours prior to testing was used during which participants studied, read, or watched movies. We ensured that the emotional content of the media participants were exposed to during the waiting period was low to avoid effects on pain ratings in the post-drug testing. At the end of each session, participants were provided with a high-protein snack and remained under supervision for a period of 10 min. 

### Plasma prolactin and amino acid levels

On the testing days, blood samples were drawn before administration of the amino acids and after the 5 hours waiting time to assess amino acid and prolactin levels. Prolactin is a hormone released by the pituitary gland with its secretion predominately regulated by dopamine (see 63 for review). Venous blood samples were collected in Vacutainer tubes coated with anticoagulant additives. Samples were centrifuged for 10 min and stored at -80 °C until assay. Plasma levels of prolactin, phenylalanine, tyrosine and other Long Neutral Amino Acids (LNAA) were determined and the ratios of phenylalanine and tyrosine to LNAA were calculated as an indirect measure of central dopamine precursor availability [57]. 

### Questionnaires

In addition to the VAS ratings after each stimulus, mood was assessed three times in each session using the Profiles of Mood States (POMS) rating scale, assessing the dimensions composed-anxious, agreeable-hostile, elated-depressed, confident-unsure, energetic-tired, and clearheaded-confused [64]. Time points of assessment were before participants’ pre-ingestion assessment of thermal sensitivity, and before and after assessment of participants’ post-ingestion thermal sensitivity.

### Statistical analysis

Two subjects were excluded from the statistical analysis because they showed inconsistent pre-ingestion ratings between the two testing days (more than 2 SD above the mean of the group).

After confirming normality (Shapiro-Wilk test), VAS ratings of perceived intensity and pleasantness/unpleasantness were analyzed with a repeated measurement ANOVA design using mixed model procedures with the factors ‘APTD’ (with the levels depleted and balanced) and ‘testing’ (with the levels pre- and post-ingestion) to test the effects of the APTD on thermal sensitivity. The same analysis was used for anxiety/calmness and mood VAS ratings. For analysis of effects on mood assessed with the POMS, raw scores were normalized to t-scores [64], and changes from morning baseline were calculated. Difference scores were entered into a 2x2 repeated measures ANOVA design using mixed model procedures with the factors ‘APTD’ (with the levels depleted and balanced) and ‘testing’ (with the levels pre- and post-pain sensitivity testing). The effects of the APTD on plasma prolactin and amino acid levels were analyzed with an ANOVA design using mixed model procedures, with the two within-subjects factors ‘APTD’ (with the levels depleted and balanced) and ‘testing’ (with the levels pre- and post-ingestion). ANOVA analyses were followed by post-hoc pairwise comparisons, calculation of generalized omega squared (ω^2^) statistics as an unbiased measure of effect sizes in ANOVA designs [48], and statistical power (1-β ) [49], when appropriate. Negative values for ω^2^ squared are treated as 0 because negative variance estimates have no meaning [50]; a value of 0.04 is the recommended minimum effect size for ω^2^, 0.25 represents moderate effects and 0.64 strong effects 

[51]. Associations between plasma levels and pain sensitivity were tested by Pearson correlations of changes in perceived intensity and pleasantness/unpleasantness of the thermal stimuli from pre- to post-ingestion with changes in plasma levels of prolactin, phenylalanine, tyrosine, or the ratios of phenylalanine and tyrosine to LNAA. The significance level was set to 5%. All statistical analyses were performed using PASW Statistics 17 (SPSS Inc. Chicago, USA).

## Results Study 2: Acute Phenylalanine and Tyrosine Depletion

### Manipulation check acute phenylalanine and tyrosine depletion

Replicating earlier results, the APTD procedure resulted in a reduction of plasma phenylalanine and tyrosine levels, indicated by an interaction of ‘APTD’ and ‘testing’ (phenylalanine F_24_=111.40, *p*<0.01, ω^2^=0.58, 1-β=1; tyrosine F_24_=274.77, *p*<0.01, ω^2^=0.76, 1-β=1). While the APTD mixture resulted in a decrease of phenylalanine (-88.77%) and tyrosine (-85.47%) post ingestion, the balanced mixture resulted in an increase (phenylalanine: 81.51%; tyrosine: 74.10%) of the two amino acids as expected. The ratios of phenylalanine and tyrosine to LNAA were significantly more decreased following the depleted amino acid mixture (phenylalanine: -90.72%; tyrosine: -89.04%) compared to the balanced amino acid mixture (phenylalanine: -41.67%; tyrosine: -39.10%; interaction ‘APTD’ and ‘testing’ phenylalanine F_24_=98.46, *p*<0.01, ω^2^=0.43, 1-β=1; tyrosine F_24_=102.44, *p*<0.01, ω^2^=0.89, 1-β=1).

Plasma prolactin levels were assessed in a subsample of eleven participants. A trend for the interaction of the within-subject factors ‘APTD’ and ‘testing’ was found (F_10_=4.73, *p*=0.06, ω^2^=0.08, 1-β=0.50). Post-hoc comparisons showed that plasma prolactin levels were increased after amino acid ingestion in the depleted compared to the balanced condition (post-ingestion: p=0.02; depleted *M*=10.34, *SD*=4.33; balanced *M*=6.73, *SD*=2.54), indicating decreased central dopamine levels.

### APTD effects on mood or anxiety

Mood and anxiety/calmness VAS ratings after each thermal stimulus were not affected by APTD within any stimulation intensity as indicated by non-significant interactions between the within-subject factors ‘APTD’ and ‘testing’ (Mood: Heat pain: F_23_=0.03, *p*=0.86, ω^2^=0, 1-β=0.05; Cold: F_23_=2.98, *p*=0.10, ω^2^=0.01, 1-β=0.38; Warm: F_23_=0.12, *p*=0.73, ω^2^=0, 1-β=0.06; Anxiety/Calmness: Heat pain: F_23_=0.14, *p*=0.71, ω^2^=0, 1-β=0.07; Cold: F_23_=1.86, *p*=0.19, ω^2^=0, 1-β=0.26; Warm: F_23_=0.33, *p*=0.57, ω^2^=0, 1-β=0.09).

The POMS mood subscale agreeable-hostile was affected by the APTD (interaction ‘APTD’ x ‘testing’ F_23_=7.26, *p*=0.01, ω^2^=0.04, 1-β=0.65). While there was no change in agreeableness in the balanced condition from pre- to post-thermal sensitivity testing (*p*=0.71), participants felt less agreeable after the sensitivity testing compared to before in the depleted condition (*p*=0.05). Other subscales of the POMS were not affected by APTD.

### APTD had no effect on perception of non-painful cool and warm stimuli

The APTD procedure did not affect perceived intensity or pleasantness/unpleasantness of the non-painful cool and warm stimuli (Interaction APTD x testing; Cool: VAS Intensity F_24_=0.07, *p*=0.94, ω^2^=0, 1-β=0.05, VAS Unpleasantness F_24_=0.63 *p*=0.43, ω^2^=0, 1-β=0.12; Warm: VAS Intensity F_24_=0.64, *p*=0.43, ω^2^=0, 1-β=0.12, VAS Unpleasantness F_24_=0.30 *p*=0.59, ω^2^=0, 1-β=0.08).

### APTD had no effect on pain sensitivity

Perceived intensity as well as pleasantness/unpleasantness of the thermal stimulation was not affected by the APTD procedure as indicated by non-significant interactions between the within-subject factors ‘APTD’ and ‘testing’ (VAS Intensity F_24_=0.88, *p*=0.36, ω^2^=0, 1-β=0.15; VAS Unpleasantness F_24_=0.23 *p*=0.64, ω^2^=0, 1-β=0.08). 

### No associations between plasma levels and pain sensitivity

Changes in perceived intensity and pleasantness/unpleasantness of the thermal stimuli from pre- to post-ingestion were not correlated with changes in plasma levels of prolactin, phenylalanine, tyrosine, or the ratios of phenylalanine and tyrosine to LNAA neither in the depleted nor in the balanced condition (all *p*’s > 0.10).

## Discussion

In the present study, neither the applied APTD nor a low dose of sulpiride had any effect on pain perception in young healthy volunteers. The results, therefore, do not support the hypothesis that dopamine has direct anti-nociceptive effects in acute experimental pain (e.g. [3-5]). 

Pain perception was assessed with different methods, including short painful heat stimuli, heat pain thresholds and tolerance, as well as temporal summation. In both studies, manipulation checks indicated that dopaminergic neurotransmission was successfully modulated but no impact on pain sensitivity was observed. Further, no associations between plasma prolactin, phenylalanine, or tyrosine levels and pain sensitivity were found in Study 2. To our knowledge, two other studies have thus far investigated the effects of dopamine agonism on human perception of experimental pain [28,29]. Treister and colleagues [28,29] tested the effect of acute administration of apomorphine on cold water immersion tolerance times, thermal and cold pain thresholds, heat pain stimuli, and conditioned pain modulation, i.e. pain inhibition of brief test stimuli induced by a simultaneously applied tonic pain stimulus. Cold water immersion tolerance times as well as conditioned pain modulation were increased by apomorphine. Interestingly, no effects of apomorphine on pain thresholds or suprathreshold pain stimuli were found [28,29], which is corroborated by the results reported here. 

The current study adds to the literature in several ways: first, sulpiride is more selective for dopamine receptors than apomorphine; the latter having also relatively high affinity for adrenergic and serotoninergic receptors [65]. Second, we show that temporal summation, at least partly reflecting the spinal cord mechanism of wind-up [66], is neither affected by increased synaptic levels of dopamine. Third, we show that lowering cerebral levels of dopamine, rather than augmenting them, also does not affect pain sensitivity in healthy volunteers, speaking against the possibility that the failure of dopamine augmentation to influence pain perception is a result of ceiling effects. Apart from the present study and the studies by Treister et al. [28,29], the role of dopamine in human pain perception has not been studied directly and the suggestion of direct anti-nociceptive effects is based on animal studies and indirect or inconsistent evidence. For example, every patient with Parkinson’s disease has impaired dopaminergic neurotransmission but not all patients demonstrate altered pain sensitivity and report clinical pain [24,67,68]. Furthermore, L-Dopa or apomorphine treatment have inconsistent effects on pain sensitivity in these patients: while some react with decreased pain sensitivity [25,69], others show no effects [70,71]. Other indirect evidence comes from studies in chronic pain patients. We and others have previously observed impaired dopaminergic neurotransmission in patients with fibromyalgia and have suggested that this contributes to their increased pain sensitivity [21,72,73]. Although dopaminergic mechanisms might differ in acute and chronic pain, treatment of fibromyalgia patients with dopamine receptor agonists have shown mixed results [74-77] (see 78 for review), and in a recent systematic review and meta-analysis, dopamine agonists received a strong negative recommendation for the treatment of fibromyalgia [79]. 

It could be argued that animal studies provide more direct evidence for anti-nociceptive effects of dopamine. Indeed, manipulations of dopaminergic functioning in rodents, usually achieved by the destruction of dopamine-rich structures such as the ventral tegmental area or intrastriatal injections of dopamine antagonists, have been shown to lead to increased, or in the case of dopamine agonists, decreased pain behaviors in response to nociceptive stimulation. However, it is interesting to note that increased pain behaviors are more consistently observed with tonic pain tests such as the formalin or writhing test compared to phasic stimuli, even if central dopaminergic neurotransmission is virtually abolished e.g. by lesioning dopamine-rich structures [11]. Stronger effects on tonic stimuli are compatible with a hypothesis of an alternative role of dopamine in pain processing we want to propose: dopamine predominantly mediates the salience of motivationally relevant stimuli, fostering coping responses instead of having direct anti-nociceptive effects. Although dopamine has been primarily implicated in the processing of appetitive stimuli, the mesolimbic dopamine system also responds to aversive and loss-related stimuli [80], potentially facilitating coping behaviors (see 81 for review). In reward processing, dopamine is crucial for incentive salience and thereby the motivation to obtain reward [82,83] (see 84 for review). In contrast, dopamine has no effect on the hedonic experience of reward [82,83].

Existing animal studies on dopamine and pain used behavioral outcomes such as tail flick or paw withdrawal latencies that are motivated behaviors in response to nociceptive stimulation. In contrast, pain perception in humans is usually assessed subjectively with verbal pain reports, describing the sensation rather than assessing motivated escape behaviors. Thus, animal and human studies assess different aspects of pain and nociceptive processing, possibly resulting in diverging findings when testing dopamine’s role in pain or nociception. The findings of the present studies as well as the results reported by Treister and colleagues (2013) that pain thresholds and pain ratings of suprathreshold stimuli as measures of pain sensitivity were not affected by dopaminergic manipulation support the idea that dopamine does not have direct anti-nociceptive effects in acute experimental pain. Interestingly, Treister and colleagues found that tolerance times in the cold water immersion task were influenced by apomorphine as would be expected if dopamine’s role in pain processing was indeed to mediate motivated behavior because pain tolerance and motivation are closely linked [85]. The observation by us and Treister and colleagues that pain ‘tolerance’ as assessed by increasing the temperature of a thermode until the highest tolerable temperature is reached was not influenced by dopaminergic manipulation does not contradict this conclusion. Increasing temperatures from a non-painful baseline are arguably more linked to sensory-discriminative aspects of pain perception and less to motivational aspects than enduring immersion of one’s hand in ice-cold water, which is more aversive and intense from the beginning. 

Other findings reported in the literature are not fully compatible with direct antinociceptive effects of dopamine, such as a positive linear relationship between the amount of pain experienced by healthy volunteers in experimental paradigms and striatal dopamine release [21,86]. If dopamine was anti-nociceptive, the reverse relationship would be expected. In contrast, higher dopamine release is compatible with higher stimulus salience and higher motivational drive associated with more painful stimuli. Further, microdialysis studies show that dopamine release peaks approximately 20 min after termination of a nociceptive stimulus [87,88]. This finding is not in line with direct pain inhibition but might fit better with the hypothesized role of dopamine in mediating the motivational value of a pain stimulus. Lastly, we have recent data that cannot be explained by pain inhibition through dopamine but by motivational effects of dopamine: in this study [89], pain-inhibiting effects of monetary wins as well as pain-enhancing effects of monetary losses were augmented by low dose sulpiride, contradicting anti-nociceptive effects of dopamine. These results suggest that dopamine biases an organism towards pain endurance or avoidance dependent on the relative importance of a pain stimulus compared to other salient stimuli. Taken together, we think that these findings support a role of dopamine in modulating the salience of a pain stimulus rather than providing evidence for direct antinociceptive effects. It should, however, be noted that we did not directly test the effects of dopaminergic manipulation on stimulus salience in the studies presented here, which should be done in future experiments. 

### Limitations

It is possible that we did not find any effects on pain sensitivity because the modulation of dopaminergic neurotransmission by standard APTD as well as 600 mg sulpiride was not strong enough and that more extreme interventions would produce altered pain sensitivity. Indeed, animal studies have typically employed non-physiological interventions such as intracerebral pharmacological manipulation or ablation of dopaminergic pathways. Further the balanced control amino acid mixtures also decreased the tyrosine to LNAA ratio. However, similar decreases with the control mixtures have been observed previously [57,62] and are not expected to yield appreciable changes in dopamine synthesis due to near saturation of the rate-limiting enzyme in dopamine synthesis, tyrosine hydroxylase [61,90-92]. Following the larger decreases produced after APTD, in comparison, dopamine synthesis and availability for release plummets [59,61,62]. Most importantly, multiple manipulation checks confirmed that dopaminergic neurotransmission was affected in both studies. Finally, we included calculations of unbiased effect sizes [48] as well as power calculations to ensure that effects were not missed because they were too small to reach significance. Both the extremely low effect sizes and the very low statistical power for the effects of the dopaminergic modulations on pain sensitivity support our conclusion that dopamine does not affect pain perception directly.

There is one result in the literature that we cannot reconcile with the proposition that dopamine mediates the salience of pain stimuli: in the recently published study by Treister and colleagues conditioned pain modulation was increased by apomorphine; i.e. volunteers rated nociceptive stimuli as less painful during a second painful stimulus under apomorphine compared to placebo [29]. However, as discussed above, apomorphine has effects on noradrenergic and serotoninergic systems [65]. Since duloxetine, a mixed serotonin and noradrenaline reuptake inhibitor, also affects conditioned pain modulation [93], the effect of apomorphine might not necessarily be mediated by the dopaminergic system. 

In summary, our results show that alterations of dopamine levels in a physiological range do not have measurable effects on pain perception in humans, suggesting that dopamine has no direct anti-nociceptive effects. Instead, we propose that dopamine’s role in the processing of nociceptive stimuli is through influences on stimulus salience and coping responses. This proposition adds to recent evidence that the dopamine system carries subjective value also of aversive stimuli.
